# Easing Restrictions During Vaccine Scarcity. How Mitigation Measures Help Tackling Associated Moral and Behavioral Challenges

**DOI:** 10.3389/phrs.2021.1604269

**Published:** 2021-10-21

**Authors:** Max Tretter, David B. Ehrlich, Ulrich von Ulmenstein

**Affiliations:** ^1^ Department of Theology, University of Erlangen Nuremberg, Erlangen, Germany; ^2^ Department of Economics and Management, Karlsruhe Institute of Technology (KIT), Karlsruhe, Germany; ^3^ Department of Law, University of Giessen, Giessen, Germany

**Keywords:** COVID-19, mitigation measures, vaccination policy, Covid-19 protective measures exemption ordinance, Covid-19 restrictions, vaccination scarcity

## Abstract

**Background:** When vaccines became first available during the Covid-19 pandemic, their demand significantly exceeded their supply. In consequence, the access to vaccines, initially, was distributed unequally. At the same time, governments started easing pandemic restrictions for vaccinated and recovered persons and restoring their freedoms since their risk of transmitting the virus is significantly reduced.

**Evidence:** We show that restoring freedoms for vaccinated and recovered persons – while upholding restrictions for the rest of the population – is morally unfair during vaccine scarcity. Further, it may yield unintended side-effects, including perverse incentives, growing rifts in society, and the expansion of marginalization.

**Policy Options & Recommendations:** We recommend accompanying easing for vaccinated and recovered individuals by mitigation measures for those who are neither vaccinated nor recovered. We propose, first, to temporarily lift the same restrictions for negative-tested individuals, as for vaccinated or recovered people. Second, the state must ensure broad and easy access to testing for everyone – free of charge.

**Conclusion:** If done right, these mitigation measures create (at least temporarily) equal access to freedom for everybody – solving the moral problem of unfair access to freedoms and counteracting possible negative consequences.

## Introduction

As pandemics emerge as a growing threat in the future, societies must be prepared to cope with recurring challenges. We focus on a challenge related to the phase in a pandemic when vaccines have become available, but the initially short supply cannot yet meet the vast demand, and not every willing person has had the chance to get inoculated yet. During this period, policymakers face special pressure to restore basic civil liberties for the immunized population while ensuring that the health care system will not be overburdened due to an overhasty return to normalcy and rapidly rising infection numbers. A common approach is to issue immunity certification to immunized individuals and ease their restrictions.

In our contribution, we examine how the unequal treatment of vaccinated and recovered individuals on the one hand, and the rest of the population on the other, is morally unfair and can produce unintended side effects. These effects include perverse incentives, growing rifts in society, and expanding marginalization. We claim that in order to prevent these negative consequences, it is crucial to introduce mitigation measures. By temporarily restoring the same freedoms for individuals who tested negative as for immunized individuals, these mitigation measures counteract inequalities in the population and thwart negative implications resulting from inequality.

## Background

Public health policies face the challenge of, first, protecting national public health, second, enabling citizens to enjoy equitable freedoms [1]. The Covid-19 pandemic illustrates this challenge: In order to protect the health of their citizens, prevent them from infecting each other, and keep national health systems from being overburdened governments worldwide have to enact freedom-restricting public health policies [2]. This challenge continues until the first vaccines that could effectively prevent transmission were approved for general use and governments began vaccinating their citizens. This marks the entry of the pandemic into a new phase, during which vaccinations are still scarce and not everyone has had the opportunity to get vaccinated. We call it, referring to the WHO [3] a transition phase.

This transition phase is of particular interest for the consideration of health care policy for several reasons. First, while high- and middle-income countries have already overcome this phase, there are still many countries in the midst of it [4]. And despite all efforts to achieve global immunization equity (COVAX), some models show that especially poorer countries will have to wait a long time for sufficient vaccination coverage [4, 5]. Second, there is a risk that the emergence of new virus variants could render current vaccines ineffective or drastically reduce their effectiveness [6], so that new vaccines would be needed—which would set the Covid-19 pandemic back to the stage of a global vaccine scarcity. Third, this transitional phase of vaccine scarcity can be viewed as a typical part of a pandemic. Given the prediction that pandemics may become more frequent in the future [7], fundamental considerations about policy options during this phase are important.

During this transitional phase and due to the perceived safety of vaccination many governments are considering to or are already easing the restrictions for vaccinated as well as for recovered individuals [8, 9] – often introducing immunity certificates for this purpose [10]. Justifications for facilitating vaccinated and recovered individuals are often based on the principle of proportionality of policy measures [11, 12]. The existing restrictions are intended to prevent individuals from transmitting Covid-19 and spreading the infection – and can be considered, legally, morally, and medically suitable, as long as they affect individuals who are able to transmit Covid-19. Further, vaccination against and recent recovery from Covid-19 significantly lower the probability to infect others [13] – especially if the basic preventive measures of wearing a medical mask in public and maintaining physical distance remain in place. Therefore, the restrictions no longer fulfill their initial purpose for vaccinated and recovered individuals – which makes easing their restrictions a mandatory consequence.

## Evidence

We apply a moral and subsequently a behavioral point of view to raise objections and illustrate problems in conjunction with easing restrictions for vaccinated and recently recovered individuals.

From a moral perspective, introducing easings for vaccinated and recovered persons is unjust when vaccine scarcity exists. The distribution of a good is only just if all persons have equal and fair access to it [14]. But as long as vaccine scarcity limits the number of people who can get vaccinated, there will be an unequal distribution of vaccinations, and some will have to wait longer than others. Where this unequal distribution follows vaccine prioritization, for which there are good reasons – e.g., higher risk of severe courses of Covid-19, higher exposure risks to the virus, or higher likelihood of transmission [15] –, and which follows moral values [16] it is fair and morally legitimate. However, where the restrictions are additionally eased for vaccinated as well as for recovered individuals it is no longer fair [17]. Instead, this would be tantamount to illegitimately withholding freedoms from people who want to get vaccinated but can’t – which would be unfair and also place an enormous burden on them [11]. This moral problem of unfair access to scarce vaccinations and subsequent easing of restrictions is further exacerbated when there are no good reasons for the intra-societally or globally unequal distribution of vaccines [4, 17].

A focal point in the behavioral framework of analyzing any public policy proposal is the assessment of potential side effects and unintended consequences. Even a well-meaning and mandatory policy can produce results contrary to its designated end. With regard to easing restrictions for vaccinated and recovered individuals, we raise several concerns that should be duly reflected in the further discourse. They are largely based on the fact that the vaccines are initially rare goods when they are newly introduced, and demand typically exceeds the short supply.

Regarding societal support for the idea of easing restrictions for the immunized population, the empirical evidence is still limited [9]. However, public and scientific opinion seems to be far from a consensus [8, 18]. According to some estimates, the part of the population opposing immunity certification – the common policy approach toward easings for vaccinated and recovered individuals [10] – might be nearly as large as the supporting part [9, 19]. A substantial amount of people appear concerned by the potential for discrimination [20] and the potential “harm [to] the social fabric of their community” [19]. These perceptions align with empirical evidence from economic experiments where we see that people are generally averse to inequity – and are even willing to destroy their own payoffs – when it exceeds a certain threshold [21].

We share the concern that – on a collective level – the introduction of easings based on immunization threatens to produce a growing rift in society between the vaccinated and recovered on the one side and the rest of the population on the other. As is evident by now, the risk associated with a Covid-19 infection is already linked to socioeconomic factors. Individuals with lower income and education levels are at higher risk of contracting Covid-19 [22] and suffering from a severe course of disease or death [23]. Even with the financial cost of vaccinations borne by the state, individual access to vaccines is, again, related to socioeconomic status [24], e.g., through differences in technological skills, internet access, personal networks, mistrust of the health care system, and susceptibility to misinformation [25]. Therefore, easing restrictions for the vaccinated could contribute to reinforcing pre-existent inequalities [26, 27], resulting in persons with higher income and better education facing lower medical risks and enjoying more freedoms. This problem exacerbates whenever immunization is required to access specific jobs [26], e.g., in care work or hospitality. In such cases, not only are pre-existing inequalities reinforced, but there is a threat of marginalization – of individuals being excluded from public, social, and working life.

Besides these implications, there are more practical challenges as well, such as the potential for fraudulent behavior [28] and the creation of *perverse incentives* for those who think themselves at very low risk for a severe course of the disease [26] to get infected on purpose to circumvent restrictions.

However, easing restrictions for vaccinated and recovered people holds vast benefits promising to clearly outweigh the concerns mentioned above. First, it generates a solid incentive to get vaccinated [29]. This incentive is especially relevant when considering a long-term perspective that incorporates subsequent phases of trying to emerge from the pandemic [30]. Besides restoring civil liberties, there are economic and mental health benefits when everyday life is gradually resumed, and restrictions vanish.

Both positive and negative implications cannot and should not be ignored. The scope and concrete implementation of easing restrictions – with a particular focus how to approach inequality in the process – will be the primary factor determining whether positive or negative effects dominate. A considerate design of mitigation measures – intended to reduce inequalities – can reduce the risk of undesired behavioral and societal side-effects.

## Policy Options and Recommendations

The transitional phase with scarce vaccines poses a challenge to public health policy: On the one hand, it is mandatory to ease the restrictions of vaccinated and recovered individuals. On the other hand, easing their restrictions is morally unfair and illegitimate and could lead to adverse behavioral implications. Nonetheless, it would be irresponsible to ease restrictions indiscriminately for all persons, regardless of whether they are vaccinated, recovered, tested, or neither, as this would risk an enormous increase in infection rates and might overburden the healthcare system [6]. Considering this situation, we propose a tiered approach with easings for vaccinated and recovered individuals and mitigating measures for non-vaccinated or non-recovered individuals during this transition phase – for which we draw inspiration from measures implemented in Germany.

When Germany was in an analogous transitional phase and at the same time recorded high infection rates, it introduced two policy measures. First, the so-called federal emergency brake [31] imposed contact restrictions and a curfew on all citizens and prohibited retail shopping and the use of personal services. Second, the *Covid-19 Protective Measures Exemption Ordinance* introduced easings for vaccinated and recovered citizens who were henceforth exempt from the above restrictions [32]. In addition, non-vaccinated and non-recovered persons had the opportunity to regain some freedoms temporarily: When showing a negative test that was no older than 24 h, people could, for example, shop at retail stores and access medical and related services while their contact restrictions and curfews remained in place [31].

With these policy measures, the German government was able to offer its citizens (at least temporary) freedoms while at the same time protecting national public health. However, although there was still a vaccine shortage, the policy measure granted more easings to vaccinated and recovered persons than negative-tested – creating unfair access to freedoms, thus losing its moral legitimacy and risking negative behavioral implications.

We propose to adopt the German approach in large parts but to adapt it so that negative-tested individuals temporarily have the same freedoms as vaccinated or recovered people. This includes lifting their contact restrictions and curfews for the 24 h following the test. To ensure that this mitigation measure is effective and does not create additional burdens for people in need [29], e.g., by requiring them to travel long distances, wait a long time, or pay a lot of money, we propose that sufficient testing capacities must be accessible all weekdays during daytime and in accessible proximity, and the cost of testing must be shared equitably between everybody [27, 33].

When implemented, these mitigation measures create equal opportunities for everybody to have their restrictions at least temporarily eased – solving the moral problem of unfair access to freedoms. On a behavioral level, these mitigation measures counteract the perverse incentives that arise from unequal access to freedom [27, 29]. In addition, they help to prevent tensions in society, as all individuals have the opportunity to enjoy the same freedoms [34] – and ensuring the wide accessibility of these tests without additional burdens contributes to preventing the expansion of social marginalization [33]. Mitigation measures could also convince the opposing part of the population [9, 19] of easings for vaccinated and recovered individuals – by addressing and resolving their concerns about potential discrimination [20] and harm to the community [19].

On the contrary, implementing these mitigation measures can lead to two problems: First, due to the accuracy of tests [34], negative-tested individuals are assumed to have a higher residual risk of transmitting Covid-19 than vaccinated and recovered individuals. Second, they could increase existing vaccine hesitancy [35]. However, first, as the remaining residual risks are not sufficient to justify the maintenance of restrictions for vaccinated and recovered individuals, it would be inconsistent to use them as an argument against temporarily restoring equal freedoms to negative-tested individuals. Plus, if tests are performed regularly and in clusters, their false-negative rate is significantly lower [34]. Second, vaccine hesitancy is not a problem during vaccine scarcity because the demand for vaccines always exceeds their supply in the transitional phase. These mitigation measures must be adjusted as soon as sufficient vaccines are available.

## Conclusion

As long as there is a scarcity of vaccines, it is morally unfair to ease restrictions for vaccinated and recovered individuals without providing opportunities for the rest of the population to regain the same freedoms temporarily. Furthermore, doing so can yield unintended behavioral side-effects. Concerns include perverse incentives, growing rifts in society, and the expansion of marginalization. To address these problems and to counteract the injustice without withdrawing previous easings, we propose the following: First, the same restrictions must be lifted for negative-tested individuals as for vaccinated or recovered people – so that the former have the opportunity to temporarily regain the same freedoms that the latter regain permanently. Second, the government must ensure extensive and easy access to testing for everyone – free of charge.
